# Proposed Interactions and Crosstalk Among Biological Aging Biomarkers (Telomere Length, Telomerase, Sirtuins, mtDNA Copy Number and mtDNA Methylation Levels) and Their Potential Role in Colorectal Cancer Risk: A Review

**DOI:** 10.3390/cells15211994

**Published:** 2026-10-31

**Authors:** Engelinah Dikeledi Macamo, Pragalathan Naidoo, Sibusiso Senzani, Khethiwe Nomcebo Bhengu, Sayed Shakeel Kader, Zilungile Lynette Mkhize-Kwitshana

**Affiliations:** 1 Department of Medical Microbiology, School of Laboratory Medicine and Medical Sciences, College of Health Sciences, Nelson R. Mandela Medical School Campus, University of KwaZulu-Natal, Durban 4001, South Africa; naidoop5@ukzn.ac.za (P.N.); bhengukn@mut.ac.za (K.N.B.); 2 Division of Research Capacity Development (RCD), South African Medical Research Council (SAMRC), Tygerberg, Cape Town 7505, South Africa; 3 Department of Biomedical Sciences, Faculty of Natural Sciences, Mangosuthu University of Technology, Umlazi, Durban 4031, South Africa; 4 Internal Medicine, Clinical Medicine, Faculty of Health Sciences, University of Witwatersrand, Johannesburg 2017, South Africa; 5 Department of Surgery, University of KwaZulu Natal, Durban 4001, South Africa; 6 Biomedical Sciences Department, School of Life and Consumer Sciences, College of Agriculture and Environmental Sciences, University of South Africa, Florida Campus, Johannesburg 1710, South Africa

**Keywords:** biological aging, CRC, telomere length, telomerase, sirtuins, mtDNA copies, mtDNA methylation, biomarker network

## Abstract

**Highlights:**

**What are the main findings?**

- Telomere length, telomerase, sirtuins, mitochondrial DNA (mtDNA) copy number, and mtDNA methylation may interact through interconnected pathways regulating biological aging.
- Accelerated biological aging may contribute to colorectal cancer (CRC) risk through genomic instability, epigenetic alterations, and chronic inflammation.

**What are the implications of the main findings?**

- Combining several biomarkers of biological aging could offer a fuller picture of how aging-related processes contribute to colorectal cancer development.
- Understanding how these biomarkers interact may guide future research on early CRC risk assessment and targeted prevention strategies.

**Abstract:**

There is a rapid increase in early onset CRC worldwide. This incidence is profoundly influenced by biological aging in the colon, yet the underlying mechanisms remain incompletely defined. This review critically evaluates evidence supporting proposed interactions among telomere dynamics, sirtuins, and mitochondrial biomarkers during colon aging and as CRC risk predictors, while highlighting areas where mechanistic validation remains limited. This insight paves the way for novel early-detection biomarkers, personalized preventive approaches, and treatments designed to directly interfere with aging mechanisms and CRC risk.

## 1. Introduction

Biological aging is a major driver of age-related diseases, presenting significant societal and economic challenges. A growing body of evidence indicates that biological aging is a major risk factor for various cancers, including colorectal cancer (CRC) [1,2,3]. While aging is marked by a gradual deterioration of cellular and physiological functions, cancer is characterized by unchecked cellular growth. Nevertheless, these two processes share multiple underlying biological mechanisms [4,5]. Among the key shared mechanisms are genomic instability, DNA damage responses, cellular senescence, chronic inflammation, mitochondrial dysfunction, and age-related changes in immune function [1]. According to GLOBOCAN 2020 estimates, CRC is a leading global health burden, responsible for approximately 1.93 million new cases and 930,000 deaths annually [6]. These figures rank it as the third most diagnosed cancer worldwide and the second deadliest [6]. This high burden is increasingly driven by a confluence of factors, including the widespread prevalence of poor dietary habits, exposure to environmental carcinogens, and poor lifestyles [7]. Biological aging elevates CRC risk through progressive functional decline, characterized by immune dysfunction, cellular damage, and molecular instability [8]. These changes stem from multiple factors, such as chronic infections, inflammation, oxidative stress, nutritional deficiencies, and socioeconomic disparities many of which also directly contribute to CRC development [9,10].

Although recent evidence points to a possible two-way interaction between biological aging processes and CRC, the precise mechanisms and the extent of this bidirectional influence remain poorly defined [11,12]. The link between cancer and biological aging stems from common molecular pathways, such as genomic instability, mitochondrial dysfunction, oxidative stress, cellular senescence, and epigenetic alterations [8,13]. CRC offers a concrete illustration of this link, since age-related alterations in these pathways could contribute to the buildup of cellular damage, disruptions in intestinal homeostasis, and the onset and progression of colorectal malignancies [8,12]. Quantifiable markers including telomere length, telomerase activity, sirtuins, mitochondrial DNA copy number, and mtDNA methylation patterns serve as measurable reflections of these shared biological mechanisms. The heightened susceptibility to CRC observed with advancing age is thought to originate from a combination of systemic biological aging and the progressive build-up of age-associated alterations within the colonic microenvironment [8]. Specifically in the colon, such alterations encompass impaired epithelial homeostasis and intestinal stem cell (ISC) function, genomic instability, epigenetic dysregulation, mitochondrial dysfunction, dysbiosis of the gut microbiome, and chronic low-grade inflammation (inflammaging) [14,15,16].

ISCs, particularly Lgr5-positive multipotent cells located at the base of intestinal crypts, depend on a specialised niche composed of neighbouring epithelial cells and subepithelial mesenchymal stromal cells for their maintenance and function [17]. These supporting cells provide signals that regulate stem cell renewal and differentiation. For example, Paneth cells secrete Wnt3, epidermal growth factor (EGF), and the Notch ligand Dll4, whereas Foxl1-positive mesenchymal telocytes produce factors such as Wnt2b, Wnt5a, R-spondin 3, and bone morphogenetic protein inhibitors, alongside certain Wnt antagonists [17,18]. This diversity of signalling molecules highlights the importance of maintaining a balance between stimulatory and inhibitory signals within the ISC niche. Age-related changes may disrupt this balance, with studies reporting reduced Wnt signalling from niche cells alongside altered Wnt responsiveness in aged ISCs, potentially impairing differentiation and normal organoid formation [15,19,20]. Furthermore, during inflammatory injury, Lyz1-positive Paneth cells may acquire stem cell-like transcriptional characteristics through Notch and SCF/c-Kit signalling, reflecting an adaptive regenerative response [21,22]. Collectively, age-associated changes in niche signalling, epithelial regeneration, and stem cell differentiation may interact with genomic and epigenetic alterations, mitochondrial dysfunction, gut microbiome dysbiosis, and inflammaging, thereby creating conditions that favour CRC development [8,16].

The pathways connecting ISC aging to colorectal carcinogenesis are probably not the same across all age groups and may vary between older and younger patients. In older people, it is thought that age-related decline of the ISC niche, combined with altered immune responses and disrupted interactions with the gut microbiota, encourages ISC senescence and compromises tissue homeostasis [23]. In younger people, a different sequence has been suggested, whereby changes in gut microbiota composition trigger excessive inflammatory responses that may contribute to CRC development, although it is still unclear whether these pathways play a causal role [24]. Additionally, under homeostatic conditions, normal ISCs sustain epithelial renewal, while colorectal cancer stem cells (CRCSCs) have the self-renewal capacity, tumour-initiating potential and maintenance properties needed to drive tumour growth [17,25]. Genetic and epigenetic changes contribute to malignant transformation, but age-related shifts in cellular function and in the surrounding microenvironment may influence how these changes are tolerated and expressed [26]. In this context, telomere length, telomerase activity, sirtuins, mitochondrial DNA (mtDNA) copy number and mtDNA methylation provide complementary, mechanistically distinct readouts of biological ageing and cellular dysfunction relevant to CRC. Combining these biomarkers with chronological age, mutational profiles and clinical features may therefore help identify distinct biological subgroups among CRC patients and explain why CRC risk varies between age groups.

CRC is one of the most common cancers tied to aging and remains a major health problem worldwide. Even though it has traditionally been thought of as a disease that affects older people, its rate has gone up in several younger age groups in recent decades [27,28]. Furthermore, most of the existing studies on this phenomenon are based on chronological aging, while biological aging may vary significantly among individuals with the same age. These trends have increased interest in figuring out how age-related molecular changes help cause CRC, and whether markers of biological aging could shed light on CRC risk, how the disease progresses, and ways to prevent it. This review critically assesses the evidence for proposed interactions between telomere dynamics, sirtuins, and mitochondrial biomarkers, while identifying areas where mechanistic confirmation is still lacking.

## 2. Biological Aging in the Colon (Local Colonic Aging)

During colonic aging the ISC homeostasis is impaired through Wnt signalling paradox leading to aged stem cells to become overly responsive to Wnt [15,16]. Thus, creating the development of abnormal, cyst like growths with dysplastic features that resemble precancerous tissue, thereby creating conditions in the colon that make it susceptible to malignant transformation [15,16]. Furthermore, impairment in the function of ISCs can disturb crypt homeostasis and weaken the integrity of the epithelial barrier. This, in turn, may foster microbial imbalance, chronic inflammation, and other alterations in the microenvironment that are associated with the development of CRC [29]. Additionally, disturbed ISC function can lead to gut barrier dysfunction manifested by decreased expression of tight junction proteins which renders the colon more susceptible to invasion by external pathogens, further hastening the aging process [30].

During biological aging in the colon, Adenomatous Polyposis Coli (APC) plays an essential role in controlling ISC balance and Wnt signalling [15]. Biological aging factors such as inflammation, cellular senescence, oxidative stress, and epigenetic drift can disrupt normal APC regulation, which may lead to compromised tissue maintenance and a heightened risk of CRC [15,31]. Current evidence indicates that a high mutational burden is not the primary driver of functional decline in the aging colon [32]. Age-related colon dysfunction in the epithelium appears to be driven predominantly by dysregulation of critical signalling pathways such as EGF and Wnt, rather than by an increased mutational burden [33]. These signalling alterations are believed to underlie many of the characteristic phenotypic changes observed during aging. Although biological aging may not be the direct cause of CRC disease, it fosters a biological environment that permits the accumulation and functional impact of somatic mutations, thereby increasing their pathogenic potential [8,34]. Most reported somatic mutations in CRC, include APC, Tumor Protein 53 (TP53), RAS, and Sma and Mad Mothers Against Decapentaplegic related protein 4 (SMAD4) [35,36,37]. Furthermore, these mutated genes can alter ISC homeostasis by disturbing pathways that control cell proliferation, differentiation, apoptotic cell death and genomic stability. Additionally, when accumulated over time, these alterations may result in stem cell failure, poor renewal of the epithelial barrier, and an increased risk of CRC [35,36].

Although there are studies reporting a disturbed ISC homeostasis [15,16,38] these studies are more based on the chronological age than the biological aging occurring in the colon. Impaired ISC can also lead to microbiome dysbiosis. There is a bidirectional relationship between the impaired ISC and microbiome dysbiosis. Microbiome dysbiosis, refers to an imbalance in the body’s natural microbial communities, which can harm normal bodily functions and play a role in the development of numerous diseases [39]. Dysbiotic bacteria cause chronic low-grade inflammation (inflammaging), which promotes cancer by driving cell growth, blood vessel formation, and blocking cell death [40]. Some studies [41,42] reported that microbial byproducts also trigger epigenetic changes that can disable tumor suppressors or activate cancer genes. Additionally, the loss of beneficial butyrate-producing bacteria weakens the gut barrier, raises colon pH, and allows bacterial products to leak into surrounding tissue [43]. In the aging of the colon, cellular senescence plays a central role. It shows up as a loss of proliferative ability in epithelial cells, a rise in DNA damage, and the production of senescence-associated secretory phenotype (SASP) pro-inflammatory signals that collectively intensify localized inflammation [32]. Thus, making the colonic microenvironment to be more susceptible to CRC. As colonic aging progresses chromosomal instability increases due to DNA damage accumulation, telomere attrition, impaired DNA repair, and oxidative stress. These alterations ultimately result in genomic instability, stem cell dysfunction, and a higher risk of CRC [8,32]. Furthermore, in the aging colon, extensive immune remodelling takes place, typified by both a decline in mucosal immunity and a heightened infiltration of immunosuppressive cells. This altered immunological landscape ultimately creates a permissive environment that enables tumors to evade immune surveillance [8].

## 3. Individual Biomarkers: Mechanisms and CRC Relevance

### 3.1. Telomere Length

In colorectal epithelial cells, where rapid turnover is essential for intestinal homeostasis, precise telomere length regulation is critical [44]. Telomeres are essential nucleoprotein structures that cap chromosome ends, preventing genomic instability [45]. With each cell division, these protective caps shorten. Once they erode to a critical point, they trigger a DNA damage response (DDR) that triggers replicative senescence or cause cell apoptosis, the latter being a fundamental process underlying biological aging [45]. Additionally, replicative senescence and cell apoptosis serve as a vital tumor-suppressive barrier by eliminating cells with compromised genomic integrity [46,47]. Dysfunctional telomere maintenance in stem or progenitor cells can lead to genomic instability, facilitating mutations in oncogenes (e.g., Kirsten RAt Sarcoma viral oncogene homolog (KRAS) and tumor suppressors (e.g., APC and TP53), thereby driving CRC initiation [48,49]. Although short telomeres are a known trigger for the genetic instability that initiates cancer, very long telomeres in healthy colon tissue are also linked to a higher risk of developing adenomas. This is likely because the extended telomeres grant newly initiated cells a higher replication ceiling, allowing them to multiply and expand into larger clones [50].

Study by Cui et al. [51] reported a U-shaped association between telomere length in peripheral blood cells and CRC risk, suggesting that having telomeres that are either too short or too long is equally associated with a heightened risk of developing CRC. Furthermore, there are limited studies associating biological aging and CRC risk using telomere length as a biomarker [8,32]. Thus far, numerous studies have shown that telomeres serve as a valuable prognostic marker and are correlated with various clinicopathological characteristics in patients with CRC [52,53,54,55]. Although these studies prove that telomere length serves as a valuable marker for CRC risk and risk factors for the development of tumors, there are limited studies that associate telomere length with the risk of CRC. The available evidence remains conflicting, with some studies reporting shorter telomere length to be associated with CRC risk [56,57,58,59]. While others reports longer telomere lengths to be associated with the risk of CRC [50,60,61,62].

### 3.2. Telomerase Activity

Telomerase functions as an RNA-dependent DNA polymerase, adding telomeric repeats to chromosome ends using its own RNA as a guide [47]. Its most basic, functional core contains only two essential parts: the catalytic protein TERT and the RNA component TERC, which supplies the template sequence. Telomerase, an enzyme that extends telomeres, is usually inactive in most somatic cells but often reactivated in cancer cells, allowing limitless replication and tumor growth [63]. Telomerase reactivation occurs in approximately 85–90% of human cancers, including 70–80% of colorectal carcinomas [64,65]. CRC therefore provides a particular example of the wider relationship linking telomere maintenance mechanisms, cellular immortality, and carcinogenesis. This reactivation enables malignant cells to bypass replicative senescence.

The presence of TERT promoter mutations in microsatellite-stable CRC highlights telomerase reactivation as a key tumorigenic event [66]. These mutations result into dysregulated TERT expression and telomerase reactivation, thereby maintaining telomere length and supporting cellular immortality [67,68]. The enzyme’s oncogenic role extends beyond telomere maintenance. Studies reported that, high telomerase activity correlates with advanced CRC stage and poor survival, while experimental TERT inhibition suppresses CRC growth [69,70,71]. In addition to its primary role, telomerase contributes to CRC in other ways, such as by protecting mitochondria and activating the Wnt/β-catenin pathway, which fosters an environment that encourages tumor growth [72,73,74,75]. These findings position telomerase as both a diagnostic biomarker and therapeutic target. Furthermore, liquid biopsy studies demonstrate circulating telomerase RNA (hTR) as a potential early CRC detection marker, while immunotherapy approaches targeting telomerase peptides show promise in clinical trials [76,77].

### 3.3. Sirtuins (SIRT1–SIRT7)

Sirtuins (SIRTs) are a class of nicotinamide adenine dinucleotide (NAD^+^)-dependent enzymes that play pivotal roles in critical processes including metabolism and inflammation to DNA repair and mitochondrial homeostasis [78,79]. The seven mammalian sirtuins, localized at different cellular compartments, form a critical network that regulates the aging process and cancer. Sirtuin dysfunction in the colon is manly implicated as a common pathway driving both inflammatory bowel disease (IBD) and colorectal tumorigenesis [80]. The reason may be that their dysregulation in the colon results into epithelial barrier disruption, dysbiosis, unchecked inflammation, and pro-tumorigenic microenvironment formation [80]. As knowledge of these sirtuins evolves, physiological and disease-related roles expand, they are increasingly recognized as a potential key to understanding the molecular bridge between aging and CRC.

#### 3.3.1. Nuclear Guardians: SIRT1, SIRT6, and SIRT7

This group of sirtuins, primarily located in the nucleus, has profound effects on gene expression, DNA stability, and stress response.

SIRT1 exhibits a complex, context-dependent duality in CRC, influencing tumor cell survival, inflammatory responses, and metabolic pathways [81]. By deacetylating key transcription factors including NF-κB, STAT3, and p53, it regulates processes such as apoptosis, inflammation, and DNA repair [81,82]. While its role in maintaining genomic stability suggests a tumor-suppressive function, its ability to inhibit apoptosis in established tumors can paradoxically support cancer cell survival.

Studies in mice reported that the depleting SIRT1 triggers both colon inflammation and changes in gut bacteria, revealing that SIRT1 loss fuels age-related gut inflammation specifically through the induction of microbiome dysbiosis [83]. The consistent downregulation of SIRT1 has been documented in both colonic biopsy specimens from IBD patients and areas of active intestinal mucosal inflammation [84,85]. Furthermore, despite its often-protective context, SIRT1 overexpression exerts pro-tumorigenic effects in CRC by activating the Wnt/β-catenin pathway, modulating miRNA expression, and enhancing Snail protein stability via JNK-dependent phosphorylation [86].

SIRT6 functions predominantly as a tumor suppressor in CRC. It is a key chromatin-bound enzyme that deacetylates histone H3 lysines 9 and 56, influencing gene expression and DNA stability [87,88]. SIRT6 influences biological aging by ensuring genomic stability, maintaining telomere integrity, and promoting metabolic balance [89,90]. In CRC, it exerts its protective effects primarily by curbing energy production through glycolysis and suppressing inflammation triggered by NF-κB [91,92]. SIRT6 deficiency leads to the suppression of R-spondin 1 (Rspo1), which is essential for the growth and renewal of intestinal epithelial cells [93]. Additionally, as reported in a mouse model, systemic SIRT6 knockout results in severe colitis and colonic mucosal erosion, while intestine-specific SIRT6 deficiency significantly exacerbates the response to DSS-induced colitis [93].

In contrast to SIRT6, SIRT7 acts as a potent oncogene in CRC. Additionally, it is a crucial regulator of cellular homeostasis, modulating essential functions like transcription, ribosome production, and proliferation [94]. Although, there are limited studies on the association between SIRT 7,colonic aging and CRC risk, researchers observed significantly higher SIRT7 levels in the colonic mucosa, alongside a notable reduction in SIRT1 in the mice [95]. Elevated expression of SIRT7 in CRC tumors, as opposed to normal tissue, drives disease progression by enhancing critical oncogenic processes including cell proliferation, migration, and metastasis [96]. Its role in promoting cellular survival during endoplasmic reticulum (ER) stress further underscores its tumor-promoting functions [97,98].

#### 3.3.2. Mitochondrial Sentinels: SIRT3, SIRT4, and SIRT5

This group operates within the mitochondria, directly controlling energy metabolism, redox balance, and cell death processes frequently hijacked in cancer.

SIRT3, the primary mitochondrial NAD^+^-dependent deacetylase, serves as a critical tumor suppressor in CRC [99]. It regulates bioenergetics, redox balance, and mitochondrial quality control [100,101,102]. SIRT3 predominantly exhibits tumor suppressive activity by limiting oxidative genomic instability and activating programmed cell death pathways [99]. The natural decline of SIRT3 with age dismantles this protective system, resulting in mitochondrial dysfunction and inflammation that drive accelerated aging [103]. Although there are lack of studies associating SIRT3, colonic aging and CRC risk, the observation of colonic SIRT3 dysregulation in one mice study [104] was linked to exacerbated colitis-like inflammation, breakdown of gut barrier defences, and enhanced tumorigenic potential. Additionally, a recent study demonstrated that SIRT3 deficiency within intestinal epithelial cells drives IL-1β production and modulates NAD^+^ metabolism via a quinolinic acid-dependent mechanism, which in turn influences T cell differentiation in both CRC and colitis [105].

SIRT4 also functions primarily as a tumor suppressor. This mitochondrial sirtuin helps control glutamine metabolism, fatty acid oxidation, and ROS balance [106,107]. It suppresses the aggressiveness of CRC by regulating E-cadherin and inhibiting glutamine metabolism [108]. Consequently, low levels of SIRT4 are strongly linked to more advanced CRC stages and a higher likelihood of disease recurrence [99,108,109]. Although there are limited studies linking sirtuins and colonic aging, dysregulation of SIRT4 have been reported to lead to excessive ECM production and intestinal fibrogenesis, a condition commonly seen in over 50% of Crohn’s disease patients [110]. Furthermore, a rat study reported that DSS-induced colitis was associated with decreased mRNA levels of both SIRT4 and SIRT1 [111].

SIRT5, however, demonstrates a clear oncogenic role in CRC. This mitochondrial enzyme catalyses the removal of succinyl modifications from lysine residues, exerting regulatory control over diverse metabolic pathways [112,113]. In CRC, SIRT5 overexpression drives growth by boosting glycolysis, glutamine metabolism, and oxidative stress resistance [114]. This role stands in stark contrast to its function in maintaining metabolic health during the aging process. Although there are limited studies associating SIRT5, colonic aging and CRC risk, at least one study reported that impaired SIRT5 activity in the colon promotes colitis through elevated PKM2 succinylation, which subsequently causes IL-1β to be overproduced and worsens inflammatory susceptibility [115].

#### 3.3.3. The Cytosolic Regulator: SIRT2

SIRT2 displays context dependent duality in CRC, mirroring its complex role in aging processes. As a predominantly cytoplasmic NAD^+^-dependent deacetylase, SIRT2 governs multiple hallmarks of biological aging through its dual regulation of structural and metabolic networks [116]. In CRC, while its p53-stabilizing function can promote tumor suppression [117,118], its metabolic plasticity (particularly via the Warburg effect) and cytoskeletal remodelling capacity can paradoxically support tumor progression [118,119,120]. Although the association of SIRT2, colonic aging and CRC risk is barely studied. Loss of SIRT2 was shown to increase colitis vulnerability by favouring pro-inflammatory macrophage differentiation, triggering NF-κB hyperactivation, and concurrently reducing tight junction protein expression [118].

This analysis demonstrates that while sirtuins collectively function as a protective network against aging, their dysregulation in CRC can transform them into either suppressors or promoters of tumorigenesis. This duality sets the stage for their critical interactions with telomeres and mitochondria, which we explore in the following sections.

### 3.4. Mitochondrial DNA Copy Number (mtDNA-CN) and Mitochondrial Methylation Levels

Mitochondrial dysfunction may act as a contributing factor to both the disruption of tight junction and cytoskeletal architecture in intestinal epithelial cells and the diminished regenerative potential of the epithelial layer [121]. Mitochondria are crucial cellular components that produce energy (ATP) via oxidative phosphorylation [122]. While this process is vital for supporting active cells, it also generates ROS as a byproduct. At low levels, ROS function as important signalling molecules for processes like immunity and cell death [123]. However, when overproduced, ROS cause oxidative stress, damaging cellular components and mtDNA [124]. This damage is linked to aging and cancer development [125,126,127].

Specifically, in CRC, mitochondrial dysfunction is a central driver of pathogenesis [128]. Major pathogenic mechanisms include metabolic rewiring, the progressive accumulation of mtDNA mutations, overproduction of ROS, and epigenetic remodelling particularly mtDNA methylation, a modification that fine-tunes mitochondrial gene transcription, DNA replication, and overall cellular bioenergetics [129,130]. The above phenomena collectively disrupt redox signalling, promote genomic instability, and create a tumor-friendly environment that enhances cancer cell survival [131]. This risk is profoundly exacerbated by age related mitochondrial decline and a reduction in mtDNA copy number. There are limited to no studies directly associating mitochondrial dysfunction, mtDNA CN and mtDNA methylation with colonic aging and CRC risk. It was shown that age-linked mtDNA defects disrupt oxidative phosphorylation (OXPHOS), instigating metabolic shifts that speed up tumor growth in the gut [130]. Furthermore, limited studies between mtDNA and CRC risk report different results. A non-linear, U-shaped relationship between peripheral blood mtDNA-CN and CRC risk was reported [132]. The results found in the study mentioned show that both reduced and elevated levels are associated with greater disease susceptibility. While increased risk of CRC was reported to be associated with high levels of mitochondrial content [133,134]. In another study by individuals with reduced mtDNA-CN in their white blood cells had a higher likelihood of being diagnosed with CRC within 15 years [135]. This association remained significant even after the researchers controlled for variables such as age, sex, smoking, BMI, blood pressure, cholesterol, and education. In addition, decreased mtDNA copy numbers was also reported in women who later developed CRC compared to the cancer free women in a Shangai study [136].

## 4. The Crosstalk Between Biological Biomarkers

Overall, this body of evidence in Table 1 shows that biological aging in the colon stems from a concurrent deterioration of telomere length, telomerase function, mitochondrial DNA integrity, and sirtuin levels. Additionally, they are considered as primary biomarkers of colonic aging and are dependently and independently associated with CRC risk. The table includes all the studies associating biomarkers with colon aging and CRC.

Several studies used cell line models to examine SIRT3 and mtDNA repair proteins [140], SIRT3 and mtROS [142], and SIRT3 and mitochondria [144]. Others investigated SIRT 6, telomerase activity and telomeres [143] and SIRT6 and TRF2 [141]. In addition, knockdown or overexpression studies have also reported mitochondrial deacetylation to be associated with oxidative metabolism, redox regulation, and chemoresistance in CRC cells, validating these molecular relationships [141,142,147]. Furthermore, rescue experiment showed that NAD^+^ restoration reversed mtDNA mutation driven intestinal aging, rescuing crypts, reducing apoptosis, and restoring stem-cell function [147]. Additionally, the animal model studies collectively show that mtDNA damage, telomere attrition, and sirtuin loss do not act independently [145,146,147]. Instead, they form a linked network where disruption in one amplifies the others, driving a pro-inflammatory, metabolic state that promotes CRC susceptibility.

Studies [135,137] reported a positive correlation between telomere and mtDNA-CN while [134] reported a negative correlation. Telomere and mitochondrial associations are only measured in mtDNA-CN and mtCN and not mtDNA methylation levels or genes related to mitochondrial dysfunction. Study Martínez et al. [139] observed shorter tumor telomeres and higher expression of SIRT1 in individuals with CRC. In addition, they observed no correlation between SIRT6 and telomere length in the tumors. SIRT3 inversely correlated with telomeres in the tumours, while SIRT1,2,3 and 4 strongly correlated in the tumour showing a clear inter-relationship among the sirtuins [34].

SIRT6 has been shown to interact with TRF2 and promote its degradation upon DNA damage [141]. Separately, quercetin was studied as an alternative means of inhibiting colon cancer cell proliferation, acting through its anticancer and epigenetic effects [143]. Although these mechanisms have been studied independently, the results imply that SIRT6-related pathways and quercetin’s epigenetic effects could provide complementary directions for future CRC treatment research. Furthermore, the studies only included SIRT6, reflecting a gap on the relationship between other SIRT 1,2,3,4,5 and 7 and telomeres/telomerase during colon aging and CRC. For sirtuins and mitochondria crosstalk all the studies reported on SIRT 3 only. The studies reported SIRT 3 as mitochondrial DNA repair regulator [140] and stress response and chemoresistance factor [142]. Finally, study by Torrens-Mas et al. [144] reported that silencing SIRT3 in SW620 metastatic colon cancer cells disrupts both mitochondrial biogenesis and function. The overall findings may suggest that sirtuins form part of a regulatory axis linking mitochondrial stress, DNA damage responses, and telomere maintenance. In CRC, this axis may become dysregulated such that telomere dysfunction and sirtuin loss no longer constrain growth but instead support survival, fuel genomic instability, and promote therapy resistance.

The studies reported in Table 1 also show the various methodological and technical challenges faced in measuring these biological aging biomarkers. Most of the studies used qPCR to measure telomeres, sirtuins and mtDNA [34,134,135,137,138,142]. Telomere length is predominantly assessed via quantitative real-time PCR or qPCR using the telomere-to-single-copy gene ratio (T/S ratio), although Southern blot-based TRF analysis remains the gold standard as it delivers absolute measurements [34]. qPCR requires strict standardisation (standard curves, well positioning, calibrator DNA) for cross-cohort comparability, whereas TRF Southern blotting demands high-quality DNA that is difficult to obtain from clinical samples and has limited sensitivity for critically short telomeres.

mtDNA copy number is quantified via qPCR by amplifying a mitochondrial gene (e.g., ND1 or COX I) relative to a single-copy nuclear reference (e.g., ALB or B2M) [134,135,137,138]. Mitochondrial methylation is typically assessed following bisulfite conversion using methylation-specific PCR (MSP) or mass spectrometry platforms such as liquid chromatography–tandem mass spectrometry (LC–MS/MS). One of major technical challenges complicating these assays is, achieving complete bisulfite conversion of the highly supercoiled, circular mitochondrial genome particularly the D-loop control region is technically demanding and prone to false-positive methylation signals [148,149].

SIRT1, SIRT3, and SIRT6 are conventionally quantified at the transcriptional level via qRT-PCR or at the protein level using Western blotting and immunohistochemistry [34,141,142,143,144]. A major limitation of these approaches is that steady state mRNA or protein levels do not correlate with enzymatic activity, given that sirtuin function is dictated by the bioavailability of NAD^+^ cofactor that declines with age and in neoplastic tissues [34]. Additionally, sirtuins are compartmentalised (SIRT3 in mitochondria, SIRT6 in the nucleus) and can translocate under stress, but bulk tissue lysis destroys this spatial context [80,146]. Therefore, static methods are insufficient. Dynamic approaches like spatial metabolomics or real time imaging are required to capture functional sirtuin activity and downstream metabolic flux [150].

### 4.1. Telomere Length/Telomerase and Sirtuins Crosstalk

Critically short telomeres act as a signal of genomic stress by activating DNA damage response pathways, particularly through p53 [151]. The activation of these pathways consumes NAD^+^, a cofactor essential for the activity of sirtuins such as SIRT1 and SIRT6 [152,153]. This depletion of NAD^+^ diminishes sirtuin function, thereby removing a critical brake that normally suppresses excessive DNA damage signalling and maintains telomere stability [154,155,156]. In CRC, the interplay between telomere shortening and sirtuin dysfunction compromises the integrity of the genome, leaving DNA damage to accumulate freely. This accelerated genomic instability, perpetuated by a cycle of escalating damage, promotes mutation accumulation and facilitates the malignant transformation of colonic epithelial cells, thereby driving CRC initiation, progression, and aggressiveness [141].

Several studies have reported that the dysregulation of sirtuins (most notably SIRT6 and SIRT1) compromises telomere stability and telomerase regulation, thereby directly promoting both genomic instability and the hallmark of cellular immortality in colorectal malignancies [141,143]. SIRT6 deficiency produces abnormal telomeres that quickly induce cellular senescence and accelerate the aging process in the colon [141]. Higher levels of SIRT1 and short telomere length was observed in individuals with CRC [139]. In addition, this may imply that though telomere shortening signals accelerated aging and high CRC risk, SIRT1 appears to be upregulated in tumors to offset this instability and enable ongoing expansion. Research on the flavonoid quercetin demonstrates that upregulation of SIRT6 markedly suppresses telomerase activity. This suppression prevents telomere length maintenance, thereby inducing telomere erosion and compelling colon cancer cells to undergo either senescence or apoptosis [143]. These findings are backed by recent review articles [8,157], which suggests that sirtuins are likely to modulate CRC risk within the colon through their actions at the telomeric and chromatin levels, where they help govern whether aging cells maintain genomic stability or instead progress toward senescence, accumulate DNA lesions, and undergo neoplastic conversion.

### 4.2. Telomeres/Telomerase and Mitochondria Crosstalk

Telomere dysfunction, triggered by shortening or shelterin protein disruption, activates p53 and initiates a comprehensive metabolic reprogramming of the cell [158]. This reprogramming is driven by p53-mediated suppression of the PGC-1α/β genes [159,160]. This suppression impairs mitochondrial biogenesis, reduces mtDNA copy number, and disrupts mitochondrial dynamics, effectively promoting aging and cancer. In situations of cellular damage, it restrains growth-related pathways such as glycolysis and the pentose phosphate pathway (PPP), thereby limiting the proliferation of compromised cells [161].

This bidirectional cycle is further intensified as mitochondria weaken, they release ROS that preferentially damage telomeric DNA due to its guanine-rich sequence [162,163]. This oxidation disrupts the protective shelterin complex, leading the cell to misinterpret the telomere as a site of DNA damage [161,164]. Furthermore, it obstructs the DNA replication process, exacerbating the natural telomere shortening that occurs with each cell division [165]. The cumulative effect forces telomeres into a critically short state, triggering cellular senescence or death. As described, mtDNA depletion represents a distinct pathway through which mitochondrial dysfunction promotes aging, independent of oxidative stress [161]. The model suggests that mtDNA depletion elevates nuclear proteins such as hnRNPA, which in turn accelerates telomere loss. A key visible manifestation of this pathway is the formation of telomere dysfunction induced foci (TIFs), with the aggregation of DNA repair proteins at telomeric sites confirming the direct molecular link to genomic instability.

CRC could be triggered or initiated by the disruption of the normal co-regulatory relationship that exists between telomeres and mtDNA [137]. However, as tumors progress to higher TNM stages, this relationship dissolves completely, revealing that the telomere–mitochondrial axis is subject to increasingly severe dysregulation throughout CRC progression [134,137]. The change in association between these biomarkers may also indicate different biological roles in different cancer stages. The telomere–mitochondrial axis is also supported by the bidirectional relationship were by telomere shortening may drive mitochondrial biogenesis as a survival mechanism to secure ATP supply, while mitochondrial changes (mtDNA-CN), in turn, can accelerate or modify the rate of telomere attrition [134]. Although there is bidirectional relationship between telomeres and mitochondria, both biomarkers can independently measure colonic aging and predict CRC. Study by Malyutina et al. [135] reported shorter telomere length and lower mtDNA to be associated with biological aging in the colon and CRC risk. Furthermore, although there are reviews supporting the findings mentioned above, the reviews cover cancer as a whole and not specifically CRC [161,166,167].

### 4.3. The Crosstalk Between Sirtuins (1–7) and Mitochondria (mtDNA-CN, mtDNA Methylation Levels)

Sirtuins maintain mitochondrial health through coordinated roles between the nucleus and mitochondria [154,168]. Nuclear sirtuins regulate gene expression for biogenesis and stress responses, while mitochondrial sirtuins directly control metabolism and oxidative defence [169]. Nuclear sirtuins, like SIRT1, function as a master regulatory command centre by deacetylating and activating transcription factors such as PGC-1α [169]. This activation orchestrates a genetic response that both stimulates the creation of new mitochondria and amplifies the expression of genes governing energy production and antioxidant protection. Concurrently, a separate group of sirtuins (SIRT3, SIRT4, SIRT5) operates directly within the mitochondria as on site managers [170]. They fine tune metabolic pathways like the TCA cycle, regulate urea cycle flux, and, most importantly, activate key antioxidant enzymes to ensure clean and efficient energy production while minimizing ROS leakage [171,172,173].

Sirtuins (SIRT1, SIRT3, and SIRT6) are NAD^+^-dependent enzymes vital for cellular resilience, yet their function collapses under mitochondrial dysfunction [155]. When oxidative phosphorylation weakens, the NAD^+^/NADH ratio drops, depriving sirtuins of the NAD^+^ required for their activity [125,174]. As a result, the protective actions of these enzymes unravel. SIRT1 can no longer drive mitochondrial biogenesis or DNA repair [174]. Additionally, SIRT3 fails to sustain energy metabolism and antioxidant defences within mitochondria [175]. Furthermore, SIRT6 loses its capacity to safeguard genomic integrity [176]. This initiates a destructive feedback cycle wherein mitochondrial impairment suppresses sirtuin activity, which in turn exacerbates the mitochondrial damage [174,175,177]. This cycle culminates in unrepairable DNA damage, elevated oxidative stress, and a state of chronic inflammation [78,178].

The relationship between sirtuins and mitochondria is bidirectional. Sirtuins preserve mitochondrial health via deacetylation and metabolic control, yet mitochondrial dysfunction in turn disables sirtuins through three primary routes NAD^+^ depletion (curbing enzymatic activity), ROS-driven inflammation (epigenetically silencing sirtuin genes), and metabolic stress (promoting DNA methylation at sirtuin promoters) [179,180]. This feedback loop holds particular significance in CRC, where metabolic reprogramming and mitochondrial failure are defining features of tumour progression. Key findings from a metabolomic analysis of UC patient biopsies include NAD^+^ depletion and altered mitochondrial morphology within the inflamed colon, pointing to an interplay between NAD^+^ metabolism, inflammatory responses, and mitochondrial dysfunction [181].

SIRT1 protects CRC mitochondria from dysfunction through its deacetylase activity, preventing the acetylation burden that would otherwise compromise mitochondrial integrity. Although there are limited studies on the crosstalk between sirtuins and mitochondria during colonic aging and CRC risk, Dong et al. [81] supports the association between SIRT1 and mitochondria by arguing that the intimate link between SIRT1 and mitochondrial dysfunction in CRC arises from its regulation of calcium homeostasis (through MCU deacetylation), its activation of PGC-1α to drive biogenesis and OXPHOS, and its influence on mitochondrial mass during resistance to chemotherapy. Unlike nuclear SIRT1, SIRT2 is context-dependent, it suppresses tumours via mitochondrial maintenance, OXPHOS, and IDH1 deacetylation, yet promotes drug resistance through ROS scavenging. In addition, the decrease in sirtuin 2 drives mitochondrial protein acetylation and mitophagy dysregulation [180].

In the colon SIRT3 directly disrupts mitochondrial biogenesis. This disruption compromises the mitochondrial pool and triggers the metabolic reprogramming essential for CRC progression [144]. Additionally, SIRT3 has been shown to regulate mitochondrial DNA repair by deacetylating key enzymes of base excision repair: NEIL1, NEIL2, OGG1, MUTYH, APE1, and LIG3 thereby establishing a direct link between SIRT3 and mtDNA maintenance in CRC [140]. In CRC, SIRT4 loss upregulatesglutaminase GLT, triggering the AKT/GSK3β/CyclinD1 axis to drive proliferation. Thus, SIRT4 suppresses tumours by limiting mitochondrial glutamine utilisation [180].

Although, there is limited documentation on how SIRT 5,6 and 7 are associated with mitochondria during colonic aging and CRC risk. SIRT5 have been reported to operate via three distinct post-translational modifications: deglutarylation, demalonylation, and desuccinylation. Its deglutarylation of GLUD1 enhances glutaminolysis, channelling carbon and nitrogen into the TCA cycle, while demalonylation of SDHA promotes succinate accumulation, stabilising HIF-1α and fostering a pseudohypoxic, pro-tumourigenic environment [180,182]. Additionally, SIRT5 deacetylates LDHB, redirecting pyruvate metabolism [182]. Collectively, these actions reprogramme mitochondrial metabolism to favour colorectal carcinogenesis. In contrast, SIRT6 exerts indirect mitochondrial effects through transcriptional regulation of glycolytic genes such as *GLUT1* and *GAPDH*, influencing the Warburg effect in CRC. Furthermore, its mitochondrial role remains less defined than that of SIRT3, SIRT4, or SIRT5. SIRT7, by comparison, is the least studied mitochondrial sirtuin in CRC, with limited characterisation of its direct mitochondrial functions.

## 5. The Interconnected Network of Aging Biomarkers Driving Colorectal Cancer Risk

**Figure 1:**
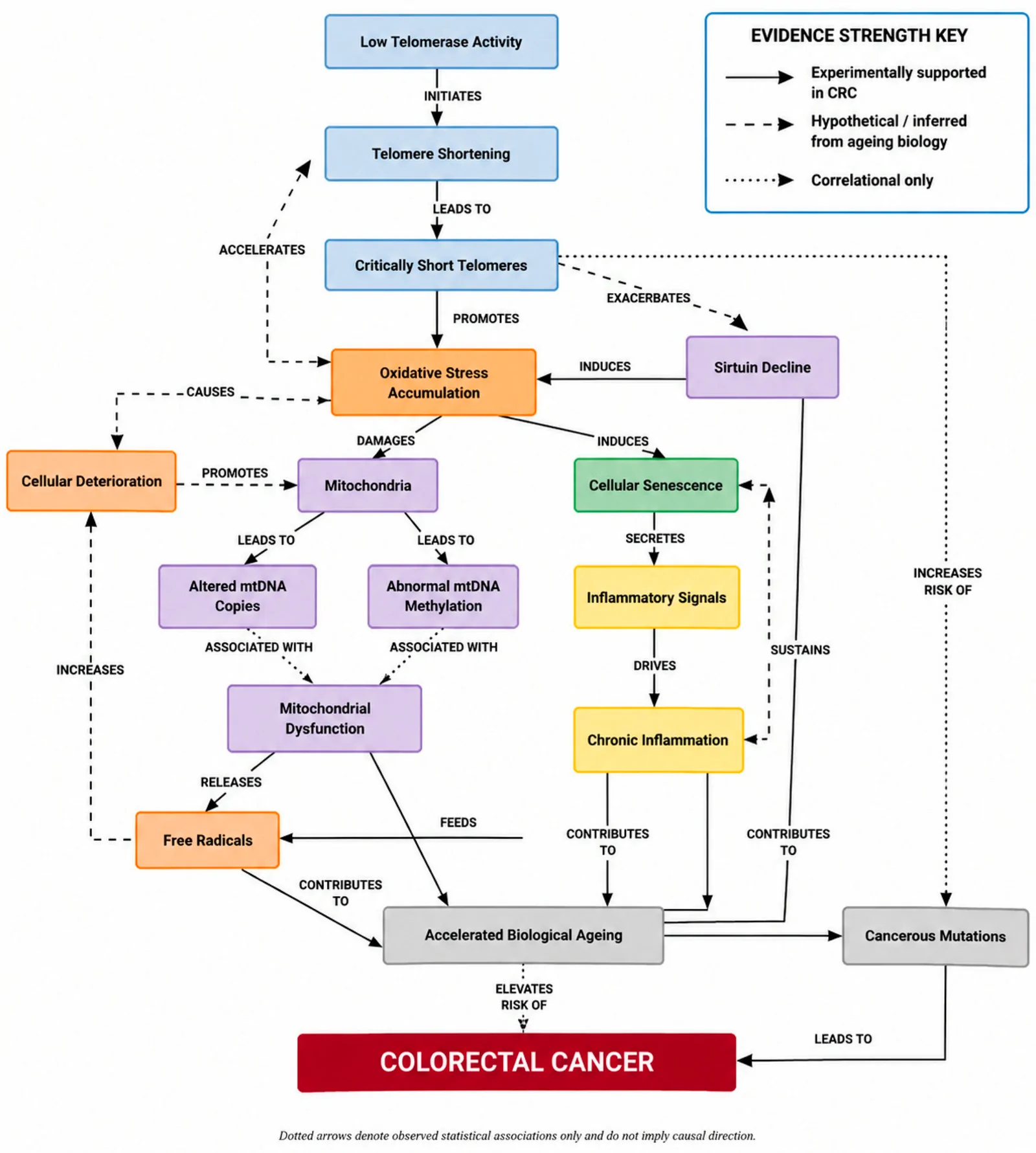
The Interconnected Network of Aging Biomarkers Driving Colorectal Cancer Risk. Created with assistance from Mermaid and Anara AI and refined by the author (Mermaid AI: https://mermaid.ai/).

Figure 1: Illustrates the core self-reinforcing feedback loops between telomere attrition, sirtuin decline, and mitochondrial dysfunction that accelerate biological aging and elevate CRC risk. The model highlights key vicious cycles: (1) Short Telomere Length and Low Telomerase Activity contribute to Cellular Senescence and Oxidative Stress; (2) Declined Sirtuins exacerbate telomere shortening and promote Mitochondrial Dysfunction; (3) Mitochondrial impairment, evidenced by Altered mtDNA Copies and Abnormal mtDNA Methylation, generates abundant Oxidative Stress, which in turn damages telomeres and induces chronic inflammation. These interconnected pathways create a state of escalating damage, where Oxidative Stress, Cellular Senescence, and Chronic Inflammation mutually reinforce one another. The collective output of this network drives accelerated biological aging and establishes a pro-tumorigenic microenvironment that promotes CRC risk.

The process of aging operates as a dynamic network of interconnected feedback loops rather than a linear sequence [183]. While potential initiators like metabolic dysfunction, genomic instability, and inflammation have been identified, no single trigger is universal [184]. Instead, the initiating event is highly personalized, where genetic susceptibility, lifestyle choices or environmental exposures can establish a specific biomarker such as eroded telomeres, dysfunctional sirtuins, or altered mitochondrial DNA (in both copy number and methylation) as the primary driver [106,185,186]. This model also applies to chronic diseases, where the distinct pathophysiology, from telomerase inhibition to mtDNA damage, determines the initial point of entry into the self-perpetuating cycle of aging and CRC development, as illustrated in (Figure 1) [187].

## 6. Master Network of Aging and CRC

Sirtuins, telomere biology, and mitochondrial function share mechanistic pathways involving ROS, p53, and PGC-1α, which supports crosstalk among them. In CRC and biological aging, this network can drive mitochondrial stress, telomere damage, and DNA-damage signalling. ROS may function as an upstream trigger and major contributor, but the relationship is likely bidirectional rather than a straightforward cause and effect cascade. According to [34], ROS promotes telomere shortening and accelerates biological aging in CRC. Accelerated telomere attrition and diminished sirtuin expression are presented as central evidence of mitochondrial dysregulation in CRC [34]. On the other hand, SIRT3 plays a protective role by limiting mitochondrial ROS production and preserving mitochondrial function, whereas SIRT6 through its interaction with TRF2 governs the DNA damage response at telomeres [141,142].

In the colon, the key connections between aging processes and cancer development involve the interplay of p53, sirtuins, telomere stability, and mitochondrial function. The tumor suppressor protein p53 acts as a critical link between the processes of aging and the development of CRC, primarily through its regulation of cellular senescence and the associated inflammatory response (SASP) [188]. In its role as “guardian of the genome,” p53 responds to cellular damage by directing cells to either repair themselves, self-destruct (apoptosis), or enter a permanent growth arrest called senescence [188]. Cellular senescence functions as a protective mechanism against cancer by putting a brake on cell proliferation. However, this benefit comes with a cost. As these aged cells build up in tissues, they release a flood of inflammatory signals (the SASP) that fuels the persistent, low-grade inflammation seen in aging [189]. While p53-induced senescence stops potential tumors from forming in the short term, it ultimately fosters a tissue environment that accelerates aging and can paradoxically increase long-term cancer risk [190,191,192].

This dual role can create a self-reinforcing cycle in which p53-induced senescence fosters a pro-tumorigenic environment. The chronic inflammation and genomic instability associated with inflammaging impair immune surveillance and increase the likelihood of cancer initiation [193]. In CRC, the frequent mutation of the TP53 gene leads to the failure of a key protective mechanism [194]. Additionally, this failure allows cells with significant genetic damage to bypass growth arrest and divide unchecked [194]. Consequently, the breakdown of p53’s regulatory and integrative functions directly facilitate colorectal tumor development [195].

Declining NAD^+^ levels reduce sirtuin function, leading to disrupted mitochondrial activity and weakened telomere protection [196]. This occurs because the sirtuins can no longer adequately support PGC-1α (which powers mitochondrial biogenesis) or maintain the antioxidant and structural defences provided by SIRT6 [197,198]. The resulting oxidative stress damages mtDNA, further diminishing energy production and amplifying inflammatory signalling [199]. At the same time, oxidative stress activates p53, a key tumor suppressor that induces senescence but also inhibits mitochondrial biogenesis via PGC-1α repression, thereby worsening the energy deficit [200,201]. P53 additionally suppresses telomerase activity, accelerating telomere attrition [200]. Although TERT can transiently alleviate mitochondrial dysfunction, its expression is largely silenced in most aging somatic cells [161,202]. Collectively, these processes create a self-reinforcing destructive loop where by a drop in cellular energy (NAD^+^) weakens protective systems (sirtuins), which leads to genetic damage (in telomeres and mitochondria) [203,204]. This damage triggers emergency signals (p53) that, ironically, worsen both the energy crisis and the genetic instability [204]. This self-reinforcing network explains why aging in the colon constitutes the strongest risk factor for CRC, as it creates a cellular environment defined by chronic inflammation, persistent DNA damage, and dysregulated signalling that collectively promote malignant transformation.

## 7. Clinical Translation of Colon Aging Research Using Biomarkers of Biological Aging

Biological aging biomarkers have demonstrated both biological plausibility and statistical association with colorectal cancer, yet the field lacks the standardised thresholds, validated multi-parametric models, diverse population validation, and prospective interventional evidence needed for clinical translation. Until these gaps are closed, the promise of precision risk stratification and personalized chemoprevention based on biological age will remain unrealised.

The clinical translation of biological aging biomarkers for CRC requires a five-stage pathway: (i) standardisation of reproducible assays for telomere length, sirtuin expression, and DNA methylation clocks [8,34]; (ii) development of multi-parametric risk models integrating these with mtDNA copy number and clinical variables [205]; (iii) creation of non-invasive screening tools including blood-based liquid biopsy, stool-based assays, and integrated risk scores for age-independent screening [8]; (iv) application of prognostic biomarkers to guide treatment notably SIRT3 as a chemoresistance predictor and KRAS–sirtuin interactions for patient stratification [142]; and finally, (v) prospective interventional trials testing sirtuin modulators, senolytics, and lifestyle modifications in populations identified by biological age scoring [143,206].

This strategy aligns with precision medicine, enabling identification of high-risk individuals for enhanced surveillance and selection of therapeutic regimens based on each tumour’s molecular aging profile [34].

## 8. Future Studies

From a biomarker perspective, the colon is not a uniform organ. The right colon, left colon, and rectum represent distinct molecular microenvironments with divergent aging trajectories [207]. Consequently, any clinically viable biomarker tool must account for anatomical subsite to ensure diagnostic and prognostic relevance. Future studies must measure colonic aging and early on-set CRC risk in the individuals below 50. Colon aging and CRC studies should also be done in sub-Saharan African regions, since most of the factors (infections, poor environmental conditions, poor lifestyle, etc.) accelerating aging and cancer are more reported in these regions. Colon health is mostly affected by the food we consume, dietary intake studies associating the type of food a population eats with biomarkers of aging should be done. This will help in recommending the type of food the population should have to prevent colon aging. CRC studies should correlate TNM stage with the biological aging biomarkers. Furthermore, a crosstalk of biological aging biomarkers should be studied in individuals without CRC, individuals with CRC and in tumours. Additionally, these studies should incorporate using different samples. This might help in understanding compartmental biology, validating surrogate markers, correlating molecular markers with clinical outcomes across tissues, linking aging mechanisms across biological levels and identifying study limitations. Finally, future studies should explore the bidirectional relationship between colonic ageing and CRC using sequencing and complementary methodologies to identify patients who may benefit from immune or disease management.

## 9. Conclusions

From the findings reported in this review we therefore propose the crosstalk between interactions and crosstalk among biological aging biomarkers (telomere length, telomerase, sirtuins, mtDNA copies, mtDNA methylation levels) and their potential risk for CRC. Additionally, there are limited studies on the relationship between CRC and biological aging. Emerging evidence, derived from the interplay of key biomarkers and mechanisms, points to a bidirectional and likely causal relationship between biological aging and CRC [208]. Consequently, molecular hallmarks of aging and cellular senescence are not only correlated with but are believed to actively contribute to CRC pathogenesis by fostering genomic instability and a pro-tumorigenic microenvironment [209,210]. Therefore, quantifying biological aging warrants further investigation as a strategy for CRC risk stratification and early detection. Finally, a deeper comprehension of these relationships is crucial for pinpointing early diagnostic markers and developing preventative interventions that disrupt aging-related pathways linked to colorectal cancer.

## Figures and Tables

**Table 1:** A summary of human and animal studies (in vitro, in vivo and clinical) reporting the possible interlink of biological aging biomarkers (telomeres, telomerase, sirtuins and mtDNA) in the colon, during CRC and as predictors of CRC risk.

Type of Study	Biomarkers	Sample Type	Methodology	Findings	Clinical Challenges	Limitations	**Ref.**
1. Prospective population-based cohort study (nested case–control design)	Leukocyte telomere length (LTL) and mtDNA-CN	Leukocyte genomic DNA extracted from peripheral blood	-15-year follow-up study of a Russian cohort (*n* = 9360, aged 45–69, 54% female) drawn from the HAPIEE study. -From the cohort, 146 CRC cases (no baseline cancer) & 799 sex and age matched controls (free of cancer & cardiovascular disease at baseline and alive through follow-up) were selected. -qPCR was used to quantify LTL and mtDNA-CN -Multivariable-adjusted logistic regression models: Confounders:(age, sex, smoking, BMI, systolic blood pressure, total cholesterol, and education).	-Shorter telomeres strongly predicted higher CRC risk (95% CI: 2.56–3.87, *p* < 0.001). -Lower mitochondrial DNA copy number also predicted higher CRC risk (95% CI: 1.54–1.89, *p* < 0.001). -Both biomarkers were independent predictors of CRC risk. -The associations remained robust when tested against reverse causation. -The two biomarkers not only mirrored biological aging but also correlated significantly with one another.	-Lacks clinically validated thresholds for LTL or mtDNA-CN that could guide interventions like earlier colonoscopy. -Both LTL and mtDNA-CN were measured as relative ratios using qPCR. In the absence of internationally standardised units cross-clinic comparisons and routine diagnostic implementation remain challenging. -Overlap in LTL distributions precludes reliable individual-level risk classification, limiting their clinical utility as standalone screening tools. -Both biomarkers show an age-related decline and are further modulated by smoking, obesity, and other modifiable lifestyle factors. -Implementing these biomarker assays on a population-wide screening scale is cost effective. -Absence of proven interventions targeting these biomarkers.	-Small sample size -Single population (same place and ethnicity) -Unmeasured confounders can affect their findings. -Both biomarkers were measured only at base point. -qPCR was the only method used.	[135]
2. Prospective cohort	Relative Telomere length (RTL) and mtDNA-CN	Peripheral blood, intestinal mucosa, and tumor samples	-A total of 166 CRC patients (diagnosed between 2010 and 2020 at Thomayer University Hospital, Prague) and 61 healthy controls were followed prospectively until March 2023. -RT-qPCR was used to quantify both RTL and mtDNA-CN.	-RTL and mtDNA-CN show their strongest correlation in early-stage CRC, which then declines as the disease advances. -Negative correlation between mtDNA-CN and RTL was evident in stage I: intestinal mucosa (ρ = −0.77, *p* < 0.0001)*,* in tumor tissue (ρ = −0.41, *p* = 0.032) and in tumor-to-intestinal mucosa ratio (ρ = −0.39, *p* = 0.046). -This relationship (initial stage) faded in later stages (II–IV), suggesting a progressive breakdown of telomere–mitochondrial homeostasis during tumour progression. -Even when controlling for age, sex, BMI, diabetes, and Tumor-Node-Metastasis (TNM) stage, higher relative mtDNA-CN in blood corresponded to a significantly decreased recurrence risk (HR = 0.43, 95% CI 0.20–0.97, *p* = 0.041). -Blood mtDNA-CN was significantly lower than tissue levels in all participants, confirming that blood and tissue biomarkers represent non-overlapping biological processes.	-A longer surveillance period would have been necessary, as the current follow-up window is too short for CRC monitoring. -The absence of a validated intervention strategy to target these biomarkers further constrains their clinical applicability. -Blood mtDNA-CN predicted recurrence but not survival. -This regulatory breakdown occurs in advanced CRC stage, precisely when effective prognostic tools are most critically needed. -Tissue biopsy is invasive and often unfeasible. -No validated clinical thresholds exist for interpretation.	-Small Sample Size and Statistical Power (when divided into different stages). -The absence of adenoma patients precludes assessment of axis changes along the mucosa adenoma carcinoma sequence, leaving the timing of dysregulation unknown. -The study measured only mtDNA-CN and RTL, leaving TERT and TFAM expression unexamined despite these being the known drivers of telomere and mitochondrial function. Thus, the data can show that the telomere–mitochondrial axis breaks down but offer no explanation for the molecular events driving that failure.	[134]
3. Retrospective case–control study	Telomere length and mitochondrial copy numbers (mtCN)	Formalin-fixed paraffin-embedded (FFPE) tissue samples	-The study enrolled 109 CRC patients, 64 cases of tubular adenoma, and 28 serrated polyp cases from Dongsan Medical Center, Keimyung University, South Korea. -Using qPCR, the study measured relative telomere length and mtCN (with COX1 as the mitochondrial gene and beta-globin as the nuclear control), while KRAS and BRAF mutations were assessed by pyrosequencing.	-mtCN was significantly lower in tubular adenomas (0.92) than in serrated polyps (1.84, *p* = 0.017), while TL showed no significant group differences. -No significant associations emerged between TL or mtCN and most clinicopathological parameters, aside from a notable elevation of mtCN in cases with vascular invasion (*p* = 0.015). Overall survival analysis yielded null results for both biomarkers (*p* = 0.483 for mtCN; *p* = 0.962 for TL). -Positive correlations between TL and mtCN were detected in normal tissue	-TL and mtCN showed no significant prognostic value for overall survival in colorectal cancer. -Invasive microdissection of tumor and adjacent normal mucosa is labor-intensive, requires specialized expertise and equipment, depends on adequate biopsy tissue, and is thus impractical for routine screening or large-scale use.	-Using FFPE samples from 1999–2003 introduces methodological challenges, as the fixation process damages DNA and biases results relative to fresh-tissue analysis. -Healthy controls were absent from the study design. -The inclusion of only 28 SPs constrained the study’s statistical power. -No multivariable survival modelling was performed. -Standardized thresholds were lacking. No universally accepted cutoff values were applied. -The study only used real time qPCR.	[137]
4. Retrospective study	Telomere length and telomerase	Tissue samples	-132 CRC participants (2006–2010); median follow-up: 5 years. -Telomere length and TERT mRNA expression were measured using Terminal restriction fragment (TRF) analysis (Telo TTAGGG kit) and real-time qRT-PCR, respectively. Telomerase activity was assessed via the TRAP-ELISA kit, with positivity defined as an OD 450 nm value of ≥0.2.	-No significant correlations: telomerase activity was not linked to telomere length groups (*p* = 0.165) or to the T/N ratio (*p* = 0.062). -Telomere length categories were not found to be correlated with TERT mRNA expression levels. -Tumors had significantly shorter telomeres than paired normal tissues (5.49 vs. 7.32 Kb; *p* < 0.001), with a strong positive correlation between the two (r = 0.685, *p* < 0.001). Rectal cancers showed the least telomere shortening compared to colon cancers (*p* = 0.021). −82.5% of colorectal cancers exhibited positive telomerase activity, no significant associations were found with gender, Dukes stage, or tumor site. -Shorter tumor telomere length (<6.35 Kb) and extreme shortening predicted better disease-free survival and zero relapses, with mean TL and Dukes stage emerging as independent prognostic factors (*p* = 0.017 and *p* = 0.008, respectively), while telomerase activity and TERT expression lacked significant prognostic value.	-Currently, no clinically validated threshold exists for utilizing telomere status as a prognostic indicator. -The primary obstacle is not an absence of biological evidence, but rather the lack of standardized protocols, consensus guidelines, and clinical validation needed to support real-world application.	-No validated clinical cut-offs for telomere length. -Inconsistent findings reported in the literature. -No established threshold for defining telomerase positivity. -Inability to perform Cox multivariate analysis for the mean T/N ratio due to an empty event subset, which prevented evaluation of its independent prognostic significance. -Absence of a significant correlation between telomere length and telomerase activity.	[138]
5. Observational case–control study	Telomere length and sirtuin expression SIRT1-7	Peripheral blood and Colorectal Tissue	-64 CRC patients and 1348 healthy controls were included. -Blood and tissue DNA telomere lengths were assessed via quantitative real-time PCR. -The analysis of SIRT1 to SIRT7 mRNA expression in tumor and normal tissues involved RNA isolation, reverse transcription to cDNA, and subsequent real-time PCR quantification. -Telomere length was correlated with fetuin-A, inflammation, redox markers, and vitamin D. -Associations with tumour site, nodal status, and survival were examined. -ANCOVA, Kaplan–Meier, and correlation analyses were used.	-CRC patients had shorter leukocyte telomeres than older controls (0.61 vs. 0.70, *p* = 0.004), despite controls being 7 years older. -Tumour telomeres were significantly shorter than normal tissue (0.43 vs. 0.65, *p* = 0.004). -PBL telomeres = normal colon > tumour (*p* = 0.003). -Telomere length declined progressively from Dukes stage A to C, but then rose again in stage D. -All seven sirtuins were downregulated in tumours (*p* < 0.001 except SIRT2 *p* = 0.003). -Tumour SIRT4 expression was lowest (0.14, IQR 0.18) and SIRT5 highest (0.55, IQR 0.51). -SIRT1 strongly correlated with SIRT2 (0.902), SIRT3 (0.509), and SIRT4 (0.758) in tumours. -Telomere length correlated with inflammation/redox markers (neutrophil-lymphocyte ratio, low fetuin-A) but not with antioxidant vitamins or trace elements. -SIRT3 inversely correlated with tumour telomere length (r = −0.353, *p* = 0.047).	-Late presentation: Many patients still present late or as emergencies, reducing curative treatment potential. -Need better molecular/prognostic markers. -Sirtuins complex; personalized therapy preferred. -Limited clinical sirtuin evidence.	-Data gaps: unmatched groups, small N, incomplete treatment data. -No fully matched cohort with both blood and tumour tissue from the same patients. -Absence of pre-neo-adjuvant biopsy samples limits conclusions about treatment effects on sirtuins. -No protein-level validation (western blot, post-translational modifications, subcellular localisation). -Ethical constraints prevented inclusion of cancer-free controls, affecting interpretation of field changes. -Tumour p53 status and patient genotyping were not assessed despite p53’s relevance to telomere/sirtuin pathways. -Methods may not have detected subtle subcellular oxidative changes. -Technical variability in telomere length quantification.	[34]
6. Cross-sectional clinical study	Telomere length and SIRT1 and 6	-Colorectal tumor tissue, -Non-tumour colorectal tissue, Subcutaneous adipose tissue (SC), Omental adipose tissue	-3 groups: 60 CRC (tumor + normal), 80 CRC (tumor + adipose), 60 obese controls (adipose only). -Telomere length was determined by TRF and qRT-PCR; the same qRT-PCR approach was used to measure relative SIRT1 and SIRT6 expression.	-Critically shortened tumour telomeres in CRC correlated with higher SIRT1 expression and indicated better prognosis. -Low SIRT1 expression (RQ ≤ 0.5) marked tumours with poorer clinical outcomes. -In non-cancerous cells, overweight/obese patients had longer telomeres than normal-weight patients. -In omental fat, obese CRC patients showed shorter telomeres than obese non-CRC controls. -No statistically significant differences in SIRT6 expression levels were observed between tumours with greater versus lesser degrees of telomere shortening.	-SIRT1 and SIRT6 can act as oncogenes or tumour suppressors depending on context, undermining their clinical utility as biomarkers or targets. -Short telomeres predict better prognosis in CRC (senescence/apoptosis) but worse in NSCLC (instability) findings are not generalisable. -Despite inflammation/oxidative stress, obese/overweight patients had longer telomeres in non-tumour cells than normal-weight individuals; mechanism unexplained. -Obese CRC patients had shorter omental telomeres than obese controls, possibly due to regional immune/cytokine activity, but this needs validation. −85% tumour telomerase positivity did not correlate with telomere length, showing reactivation alone does not drive CRC telomere dynamics.	-A larger cohort is needed to properly investigate sirtuin telomere obesity cancer relationships. -Unable to separate independent effects of obesity and CRC. -Tumur telomere length and T/N ratio did not correlate with Dukes, TNM, recurrence, or differentiation. -No association with any clinicopathological variable; no useful cutoff identified. -Neither SIRT1 nor SIRT6 expression correlated with BMI subgroups. -Shorter adipose telomeres in obese CRC vs. obese controls need mechanistic explanation. -Telomere prognostic value may be tumour-type specific; findings may not extend beyond CRC.	[139]
7. Experimental in vitro laboratory study	SIRT3 and mtDNA repair proteins	HT-29 human colon cancer cells	-Six mitochondrial base-excision repair proteins (NEIL1, NEIL2, OGG1, MUTYH, APE1, LIG3) were purified, incubated with SIRT3 and NAD^+^, and assessed for deacetylation using ELISA measuring acetyl-lysine.	-Sirt3 deacetylated all six proteins, with the most pronounced deacetylation occurring only when both Sirt3 and NAD^+^ were present, confirming that the effect was dependent on Sirt3 enzymatic activity. -This suggests that SIRT3 can regulate mitochondrial DNA repair in colorectal cancer by modulating mtBER protein function, which could influence CRC risk.	-Findings are restricted to HT-29 cells showing Sirt3 deacetylation of mtBER proteins; no patient outcomes or treatment benefit demonstrated. Further research needed to map full interactions and clinical relevance. -Dual role of SIRT3 can act as oncogene or tumour suppressor depending on context, making it difficult to develop as a simple therapy target. -Reducing mtBER efficiency may enhance chemosensitivity, but this same system protects cells from oxidative DNA damage so targeting it could have adverse effects.	-Experiments used purified proteins and HT-29 cells; no patient or animal data, so clinical translation is not demonstrated. -Reliance on one colon cancer cell line and controlled assay conditions may not reflect CRC diversity. -Authors acknowledge further research is needed to fully explain the SIRT3-mtBER interaction.	[140]
8. Hybrid of in vitro molecular biology and ex vivo clinical pathology	SIRT6 and TRF2 (a telomere-binding protein)	Human cancer cell lines (HeLa, HCT116)	**In Vitro Models (Cell Cultures):** -Experiments were performed on human cervical (HeLa) and colorectal (HCT116) carcinoma cell lines. -Both non-transformed (BJ-hTERT) and oncogene-transformed (BJ-EHLT/RasV12) human fibroblasts were employed to represent different transformation states. -Replication stress was provoked by treating cells with genotoxic compounds, specifically camptothecin (CPT), hydroxyurea, and cisplatin. **In vivo Clinical Data (Patient Samples):** -Tissue microarrays were constructed from 135 colon and 50 rectal specimens to enable IHC-based quantification of SIRT6 and TRF2 protein levels directly within tumor tissues. **Molecular & Biochemical Assays:** -Direct binding between TRF2 and SIRT6 was demonstrated using pull-down assays with GST-tagged TRF2 and His-tagged SIRT6, along with co-immunoprecipitation (co-IP) experiments. -MS/foot printing identified TRF2 K176/K179/K190 as SIRT6 deacetylation sites. -siRNA knockdown of SIRT6 and PARP1 assessed their roles in TRF2 stability and DDR -Drug sensitivity (colony formation) and apoptosis/cell cycle (flow cytometry with Annexin V) were quantified.	**SIRT6-TRF2 Interaction and Degradation** -SIRT6 associates directly with the TRFH domain of TRF2, and this interaction occurs independently of DNA. -Treatment with camptothecin (CPT) substantially increases the co-precipitation of SIRT6 with TRF2. -The injury-induced rise in SIRT6-TRF2 binding is eliminated by PARP inhibitors (*p* < 0.05). -Knockdown of SIRT6 markedly prolongs TRF2 protein half-life, confirming that SIRT6 is essential for TRF2 turnover (*p* < 0.01 at 24 h of cycloheximide treatment). **Mechanistic Post-Translational Modifications** -SIRT6 removes acetyl groups from TRF2 at three specific lysine positions: K176, K179, and K190. -This deacetylation event initiates ubiquitin-dependent proteasomal degradation of TRF2; loss of SIRT6 results in a global decrease in ubiquitinated TRF2 species. **Clinical Correlation in Colorectal Cancer (CRC)** -In a cohort of 185 CRC patients, SIRT6 and TRF2 protein levels showed a highly significant inverse relationship (*p* < 0.0001). -Among samples with low TRF2, 78% exhibited high SIRT6 expression; conversely, in tumors with high TRF2, 70% displayed low SIRT6. -These clinical data support the notion that SIRT6 acts as a tumor suppressor in colorectal cancer by driving the degradation of the oncogenic TRF2 protein.	-Although the SIRT6-TRF2 interaction is mechanistically attractive, its full contribution to DNA repair and apoptosis pathways remains unclear. The authors concede that these functions “remain to be fully elucidated,” precluding immediate clinical targeting. -The biology may extend beyond a straightforward SIRT6-to-TRF2 cascade. The study cannot exclude the participation of other regulatory proteins, such as Siah1, suggesting that additional layers of control may influence this axis. -The inverse correlation observed in the CRC cohort is compelling but does not yet confirm that modulating this pathway will improve therapeutic outcomes across all patients. Further prospective studies are required to establish clinical utility.	-The authors concede that the precise function of the SIRT6-TRF2 interaction within the broader DNA damage response and apoptosis networks has not yet been definitively characterized. -The study cannot discount the participation of other proteins, notably the E3 ligase Siah1, in TRF2 degradation implying that the proposed pathway may be embedded within a more elaborate regulatory system. -Although a robust inverse relationship between SIRT6 and TRF2 was observed in patient tissues, this association does not constitute proof that manipulating these protein levels would directly inhibit tumor progression in a clinical setting.	[141]
9. A translational research study integrating in vitro cell line experiments with immunohistochemical analysis of patient-derived tissue samples.	SIRT3 and mitochondrial ROS (mtROS)	Cell lines (HT29, HCT116, SW480, and Caco2) and FFPE tissue samples	-shRNA and siRNA were used to knockdown SIRT3, SOD2, and PGC-1α in CRC cells. -MitoSOX Red was used to measure mtROS. Apoptosis was assessed using Annexin Vstaining. Chemosensitivity with CCK-8 assays was analysed. -NAD^+^/NADH ratios, ATP levels, and lactate production were also analysed. -Clinical samples were used for immunohistochemical (IHC) scoring to quantify SIRT3 protein levels, and these scores were subsequently correlated with patient survival outcomes.	-SIRT3 expression emerged as a critical factor driving chemoresistance in CRC cells. It protects cells from apoptosis through two main pathways: activating SOD2 to eliminate drug-triggered mtROS and modulating PGC-1α to preserve mitochondrial function. -Silencing SIRT3 led to reduced mitochondrial bioenergetics evidenced by lower ATP levels and a diminished NAD^+^/NADH ratio along with a marked shift toward anaerobic glycolysis. -At the clinical level, elevated SIRT3 protein in tumor tissues was strongly correlated with poorer cancer-specific survival, establishing SIRT3 as an independent prognostic indicator.	-SIRT3 promotes chemoresistance in CRC by activating SOD2 to quench drug-induced ROS, thereby blocking apoptosis, and by upregulating PGC-1α to enhance mitochondrial metabolism (ATP and NAD^+^/NADH). -High SIRT3 in CRC tissue independently predicts worse cancer-specific survival, marking it as a poor prognostic biomarker. -Chemotherapy-induced oxidative stress can trigger a compensatory rise in SIRT3 and PGC-1α, which boosts mitochondrial metabolism and further shields cancer cells from drug effects creating a vicious cycle that reinforces resistance.	-The chemotherapy-treated subgroup was severely underpowered (n = 13) compared to the untreated group (n = 142), undermining the reliability of comparative analyses. -The proposed mediator FOXO3 remains speculative, as the study failed to experimentally verify the specific transcription factor deacetylated by SIRT3 to regulate SOD2 and PGC-1α. -High SIRT3 strongly predicted poorer cancer-specific survival but showed no significant link to recurrence-free survival, suggesting its prognostic value does not extend to relapse risk. -Western blot confirmation of IHC findings relied on only 13 frozen CRC samples, limiting the robustness of the protein-level correlation.	[142]
10. in vitro experimental cell-culture study	SIRT6, telomerase activity and telomeres	L132: normal epithelial cells HCT116: primary colon cancer cells COLO32: primary colon cancer cells COLO205: metastatic colon cancer cells	-Cells were grown in DMEM with 10% FBS and antibiotics, then treated with quercetin at 80 µM and 120 µM for 72 h. -A range of quercetin concentrations (20, 40, 80, 120, 160, and 320 µM) was tested, and CCK-8 assays were used to measure viability and derive IC50 values. -Cellular morphology was examined at 24, 48, and 72 h using an inverted phase-contrast microscope. -Anti-aging enzyme activities specifically collagenase, elastase, and hyaluronidase were evaluated. -Levels of SIRT6, Klotho, cytochrome c, proteasome 20S, and telomerase were measured using ELISA-based methods. -Relative telomere length was determined via quantitative PCR (qPCR). -miRNA expression patterns were characterized, followed by pathway analysis to identify relevant biological networks.	-Quercetin inhibited cell growth in a dose responsive manner; IC50 values at 72 h were 80 µM for L132, COLO320, and COLO205, and 120 µM for HCT116. -Cancer cells exhibited apoptosis-associated morphological alterations, while normal L132 cells remained largely unaffected. -SIRT6 protein levels increased significantly in HCT116, COLO320, and COLO205 (*p* = 0.001, *p* < 0.001, and *p* = 0.004, respectively, varying by dose and cell line). -Klotho expression rose significantly in HCT116 and COLO320 (*p* = 0.001 and *p* < 0.001). -Proteasome 20S levels decreased significantly in HCT116 and COLO320 (*p* = 0.009 and *p* = 0.01). -Collagenase activity was markedly inhibited at 120 µM in HCT116, COLO320, and COLO205 (each *p* = 0.001). -Quercetin reduced telomerase activity by 93% (*p* < 0.001) and shortened telomere length by as much as 75% (*p* < 0.01) in treated colon cancer cells.	-This is an exclusively cell-based investigation, not a patient derived study, meaning the observed effects cannot be assumed to translate directly to humans with colorectal cancer. -The use of only four cell lines (L132, HCT116, COLO320, and COLO205) may not capture the full biological diversity and genetic complexity of clinical CRC. -No human outcome measures, dosing schedules, or toxicity/safety assessments were performed, so the study does not yet support any clinical recommendations for quercetin use.	-This is exclusively a cell line study, lacking in vivo or clinical validation. Consequently, factors such as bioavailability, metabolism, and organ distribution in a living system are not accounted for. -The use of only four human cell lines (one normal and three CRC, representing primary and metastatic stages) cannot fully capture the genetic and epigenetic heterogeneity of patient tumors. -While the study correlates increased SIRT6/Klotho and decreased telomerase with quercetin treatment, it does not use inhibitors or knockdown approaches to prove that SIRT6 elevation is the direct cause of cell death. -The 72-h exposure period may not reflect longer-term biological consequences or the development of resistance mechanisms over time. -The miRNA expression profiling and pathway analysis offer extensive theoretical predictions, but these were not experimentally validated using functional assays in this study.	[143]
11. in vitro laboratory experimental study	SIRT3 and mitochondria	SW620 human metastatic colon cancer cell line,	-RT-qPCR –measured biogenesis(*pgc-1α*, *nrf1*, *tfam*, *mtssb*, *sirt1*) and functional (*coxiv*, *atpase*, *ucp2*, *ucp5*, *idh2*) genes. -Western blotting—quantified SIRT3, metabolic enzymes (IDH2, PDH, LDHA), biogenesis factors (PGC-1α, TFAM), uncoupling proteins (UCP2, UCP5), and all OXPHOS complexes. -Mitochondrial function—assessed via COX/ATP synthase/LDH activities (spectrophotometry), respiration (Oxygraph), superoxide (MitoSOX), membrane potential (TMRM), and confocal imaging (MitoTracker/LysoTracker). -Cancer progression evaluated by migration (scratch, 60 h), clonogenicity (1500 cells, 14 d), and viability (crystal violet). -Migration (scratch, 60 h), clonogenicity (1500 cells, 14 d), and viability (crystal violet) assessed SIRT3’s role in cancer progression.	-Knockdown validation: SIRT3 mRNA decreased by 69% and protein by 47%, confirming efficient silencing. -Biogenesis collapse: mRNA for *pgc-1α* (−63%), *nrf1*, *tfam*, *mtssb*, and *sirt1* all significantly reduced (*p* < 0.001); protein levels of PGC-1α and TFAM were also lowered. -Metabolic gene/protein dysregulation: *coxiv*, *atpase* (−35%), *idh2* (−39%), *ucp2*, and *ucp5* decreased at mRNA level; protein UCP2/UCP5 fell ~20%, while IDH2 and PDH were reduced but LDHA remained unchanged. -OXPHOS complex decline: Complex IV showed the largest drop (−50%), Complexes I, III, and V were significantly reduced, but Complex II was unaffected. -Enzymatic and respiratory shifts: COX activity fell 22% (*p* < 0.05), ATPase showed a non-significant downward trend, whereas LDH activity surged > 50% (*p* < 0.01), indicating a glycolytic shift; both basal and maximal oxygen consumption rates were reduced. -Oxidative stress and membrane potential: mitochondrial superoxide (MitoSOX) increased by 92% (*p* < 0.01), and membrane potential (TMRM) was significantly lowered. -Morphological and functional outcomes: SIRT3-silenced cells exhibited mitochondrial clustering/aggregation rather than a normal dispersed network, abnormal lysosome distribution with minimal colocalization, and reduced migratory capacity in wound-healing assays.	-Paradox: SIRT3 exhibits context-dependent dual roles (oncogene vs. tumour suppressor) depending on tissue type and stage, complicating therapeutic targeting. -Chemoresistance: High SIRT3 activity sustains oxidative metabolism, supporting tumour cell survival under stress and during treatment, and may promote metastasis. -ROS balance: SIRT3 fine-tunes ROS at pro-proliferative levels; its loss drives excessive ROS that can trigger apoptosis, creating a narrow therapeutic window. -Metastatic potential: Functional mitochondria are required for migration; SIRT3 silencing reduces migratory capacity, suggesting anti-metastatic benefit, but requires careful dosing to avoid widespread metabolic harm. -Therapeutic opportunity: SIRT3 knockdown compromises mitochondrial integrity and cancer cell viability, presenting a potential colon cancer strategy, yet demands selective inhibitors that spare normal tissues. -Metabolic flexibility: Cancer cells compensate via glycolysis (evidenced by increased LDH activity after SIRT3 loss), which could counteract therapies aimed at mitochondrial disruption and drive resistance.	-SW620 only; limits generalizability to other stages or colon cancer lines. -No animal models (xenografts) to validate effects in a physiological tumour microenvironment. -Off-target effects not ruled out. -No pharmacological inhibitors. -Narrow functional scope, only migration and clonogenicity. -No unbiased omics. -Activity increased but protein unchanged; mechanism unexplained. -*t*-test only, no multiple-testing correction. -No patient tissue validation. -Morphology assessed visually, not quantified.	[144]
12. Experimental preclinical study	Telomeres and telomerase	Mouse colonic and intestinal tissue Genetically modified mouse stem cells Mouse-derived intestinal enteroids Human colonic biopsies Colon-cell model	-Used histology and colonoscopy to evaluate cryptitis, crypt abscesses, epithelial damage, and colonic inflammation. -Quantified DNA damage via γH2AX immunostaining. -Characterized immune-cell populations using flow cytometry. -Quantified DNA damage via γH2AX immunostaining. -Characterized immune-cell populations using flow cytometry. -Quantified DNA damage via γH2AX immunostaining. -Characterized immune-cell populations using flow cytometry. -Quantified DNA damage via γH2AX immunostaining. -Characterized immune-cell populations using flow cytometry.	-In late-generation mice, dysfunctional telomeres caused colonic degeneration, crypt damage, and increased DNA-damage signalling. -Telomerase restoration in Lgr5^+^ cells reduced inflammation and damage. -YAP1 was central. -Telomere dysfunction activated YAP1, increasing binding to IL-18 and inflammasome genes. -YAP1 depletion reduced pro-IL-18. -Microbiome amplified inflammation. -Antibiotics reduced inflammatory markers. -Microbes mediated pro-IL-18 maturation. **Human Evidence** -Telomeropathy biopsies had elevated γH2AX, YAP1, and IL-18 vs. controls.	**Therapeutic Implications** -YAP1, IL-18, caspase-1, and telomerase restoration are potential intervention targets. -Clinical translation remains premature. -The study frames YAP1 or IL-18 inhibition as candidate approaches for intestinal inflammatory disorders, not established therapies. **Key Barriers** -Small, highly selected human cohort. -Pathway needs validation across broader patient groups and disease stages. -Cell-specific telomerase restoration is challenging. -Systemic telomerase strategies require careful evaluation in CRC-prone populations due to context-dependent effects in regeneration and cancer.	-Only 3 telomeropathy biopsies and 10 controls limited statistical power and external validity. -Lgr5-Cre system produced mosaic, regional epithelial reactivation. Incomplete rescue and persistent systemic ageing likely explain the modest survival benefit. -Targeting only epithelial Lgr5^+^ cells left inflammatory stromal cells unaffected. Broader epithelial or stromal reactivation models are needed. -The study established a mechanism for telomere associated colonic inflammation but did not test adenoma formation, CRC incidence, metastasis, or therapy response.	[145]
13. Primary mechanistic preclinical study with translational validation	SIRT3 and mitochondrial protease (LONP1)	APC-driven mouse model Tumour and adjacent normal tissue samples SW480, AGS, Hela, HEK293T, and U87 cells	-Intestinal epithelium-specific SIRT3-deficient ApcMin/+ (AVS) mice knockout model was created by crossing ApcMin/+; Villin-Cre with SIRT3fl/fl (AVS) mice. -Colorectal tumour tissues with matched adjacent non-tumour tissues using Western blotting and IHC to evaluate the expression levels of SIRT3 and LONP1 and examine their correlation. -LONP1 deacetylation sites were characterized by mass spectrometry, whereas cellular metabolic activity was measured using Seahorse extracellular flux analysis.	-**In vivo**: Intestinal SIRT3 KO increased adenoma number, size, PCNA, β-catenin, and COX-2 in APC-driven tumours. -**In vitro**: SIRT3 loss raised LONP1 expression and acetylation in SW480 cells and mouse tumour tissue. -**Functional effects**: Acetylation-mimic K145Q enhanced OXPHOS, ATP, proliferation, and migration; deacetylation-mimic K145R reversed these effects. -**Tumour growth**: K145Q formed larger xenograft tumours with higher ATP; K145R reduced tumour progression and ATP content. **-NAD^+^ rescue**: NAD^+^ restored SIRT3 activity, reducing LONP1 expression and acetylation; nicotinamide blocked this effect.	-SIRT3 and LONP1 show promise as mechanistic targets in colorectal cancer, but current evidence is insufficient to support their use as clinical biomarkers of biological ageing or CRC. -The role of SIRT3 appears to be context-dependent, and targeting mitochondrial metabolism systemically may have unintended effects on healthy tissues. -Although NAD^+^ supplementation decreased LONP1 acetylation in vitro, there is no clinical evidence demonstrating that it prevents or treats CRC in humans. -Direct inhibition or knockout of LONP1 is not a feasible therapeutic strategy, as complete LONP1 loss is embryonically lethal. -Modulating LONP1 expression or the SIRT3–LONP1 signalling pathway may represent a safer and more practical therapeutic approach for CRC.	-Lesions develop mainly in the small intestine, so the model is intestinal not strictly colon specific. -SIRT3 shows divergent associations across cancers and stages, making a universal tumour-suppressor interpretation premature. -Findings need confirmation in additional tumour cell lines beyond gastrointestinal-focused experiments. -Human CRC tissue and proteomic data show altered SIRT3/LONP1, but the study does not establish diagnostic thresholds, prognostic performance, or predictive value for therapy.	[146]
14. Primary mechanistic preclinical ageing study	mtDNA NAD^+^ and telomeres	Tissue, organoids and stem cell reporter samples	-PolgA mutator mice with graded mtDNA burdens were studied at 3, 8, and 12 months. -Intestinal aging was assessed by histology, apoptosis, p21, telomere length, and organoid formation. -mtDNA mutations, NAD^+^/redox state, and ISC markers (LGR5, Wnt, β-catenin, Cyclin D1) were quantified. -Mechanistic studies included RNA-seq, ISR/UPRmt protein analysis, ISRIB inhibition, Atf5 knockdown, and nicotinamide mononucleotide (NMN) supplementation in vivo and in organoids.	-High mtDNA burden caused fewer crypts, longer villi, more apoptosis, elevated p21, shorter telomeres, and reduced organoid formation. -Crypt cells showed lower membrane potential, higher ROS, elevated NADH/NAD^+^ ratio, and depleted NAD^+^. -High mtDNA burden reduced Wnt/β-catenin signalling and LGR5^+^ ISCs. NMN restored both. -ATF5-dependent UPRmt mediated the effect: mtDNA mutations activated ISR and ATF5; ISRIB or Atf5 knockdown improved organoid growth. -LONP1 was selectively increased with high mtDNA burden and reduced by ISRIB or Atf5 inhibition. -NMN improved crypt architecture, apoptosis, and organoids but did not restore mitophagy or reduce mtDNA burden.	**Potential Markers (Unvalidated)** -mtDNA mutation burden, NAD^+^ status, and LONP1 are candidate markers of intestinal biological ageing. -None is clinically validated for screening or risk stratification. **NMN Findings (Preclinical Only)** -NMN was beneficial in a short-term mouse intervention. -Does not establish human dose, duration, long-term safety, or efficacy. **Safety Concerns** -NAD^+^ augmentation safety is unknown in individuals at elevated cancer risk. -NAD^+^ metabolism affects both normal stem-cell regeneration and tumour metabolism. **CRC Relevance** -Supports the mtDNA mutation, NAD^+^, mitochondrial stress intestinal stem-cell axis. -Requires colon-specific primary studies before linking to colorectal tumorigenesis.	-The study examines small-intestinal ageing and cannot directly establish effects on colonic ageing, colonic crypts, adenomas, or colorectal cancer. -While the authors note that mtDNA mutations accumulate in aged human intestinal tissue, they did not conduct a new human cohort, biopsy validation, or clinical intervention study. -Several core mouse experiments used only three mice per group, limiting statistical precision and the ability to detect biological variability. -The high-burden Polg model produces accelerated, systemic mitochondrial ageing that may not replicate the timing, mutation spectrum, or tissue specificity of normal human aging. -NMN improved tissue and stem-cell phenotypes but did not correct mtDNA mutation burden or defective mitophagy.	[147]

## Data Availability

The original contributions presented in this study are included in the article. Further inquiries can be directed to the corresponding authors.
